# Prevention and control of rheumatic fever and rheumatic heart disease: the Cuban experience (1986–1996–2002)

**Published:** 2008-06

**Authors:** Porfirio Nordet, Raimundo Lopez, Luis Sarmiento, Porfirio Nordet, Alfredo Dueñas

**Affiliations:** World Health Organisation, Geneva, Switzerland; Pinar del Rio Teaching Hospital, Pinar Del Rio, Cuba; Pinar del Rio Teaching Hospital, Pinar Del Rio, Cuba; Cuban Institute of Cardiology, Havana City, Cuba; Cuban Institute of Cardiology, Havana City, Cuba

## Abstract

**Background:**

Rheumatic fever (RF) and rheumatic heart disease (RHD) are still major medical and public health problems mainly in developing countries. Pilot studies conducted during the last five decades in developed and developing countries indicated that the prevention and control of RF/RHD is possible. During the 1970s and 1980s, epidemiological studies were carried out in selected areas of Cuba in order to determine the prevalence and characteristics of RF/RHD, and to test several long-term strategies for prevention of the diseases.

**Methods:**

Between 1986 and 1996 we carried out a comprehensive 10-year prevention programme in the Cuban province of Pinar del Rio and evaluated its efficacy five years later. The project included primary and secondary prevention of RF/RHD, training of personnel, health education, dissemination of information, community involvement and epidemiological surveillance. Permanent local and provincial RF/RHD registers were established at all hospitals, policlinics and family physicians in the province. Educational activities and training workshops were organised at provincial, local and health facility level. Thousands of pamphlets and hundreds of posters were distributed, and special programmes were broadcast on the public media to advertise the project.

**Results:**

There was a progressive decline in the occurrence and severity of acute RF and RHD, with a marked decrease in the prevalence of RHD in school children from 2.27 patients per 1 000 children in 1986 to 0.24 per 1 000 in 1996. A marked and progressive decline was also seen in the incidence and severity of acute RF in five- to 25-year-olds, from 18.6 patients per 100 000 in 1986 to 2.5 per 100 000 in 1996. There was an even more marked reduction in recurrent attacks of RF from 6.4 to 0.4 patients per 100 000, as well as in the number and severity of patients requiring hospitalisation and surgical care. Regular compliance with secondary prophylaxis increased progressively and the direct costs related to treatment of RF/RHD decreased with time. The implementation of the programme did not incur much additional cost for healthcare. Five years after the project ended, most of the measures initiated at the start of the programme were still in place and occurrence of RF/RHD was low.

## Summary

Rheumatic fever (RF) and rheumatic heart disease (RHD) are still major medical and public health problems mainly in developing countries. These diseases affect an estimated 15 million people worldwide, with more than 200 000 deaths annually and hundreds of thousands of people disabled. There are roughly 2.4 million children of school-going age (five- to 14-year-olds) who are prematurely and severely affected by these diseases, one million of which live in sub-Saharan Africa. Millions of people are unaware of having the disease and are not receiving secondary prophylaxis.^1^-^15^

Pilot studies conducted during the last five decades in developed and in developing countries indicated that the prevention and control of RF/RHD is possible.^14^-^24^ In Cuba during the 1970s and 1980s, epidemiological studies were developed in selected areas to determine the prevalence and characteristics of RF/RHD, and several long-term strategies were tested for prevention of the diseases.^20^-^23^,^25^-^28^ In 1986, the incidence of RF was between 10.5 and 34.3 cases per 100 000 school children and the prevalence of RHD was between 0.2 and 2.9 per 1 000 school children.^22^ Pinar del Rio is a Cuban province that had one of the highest incidences of RF, and the prevalence and severity of RHD required large-scale hospitalisation and cardiac surgery.^22^,^23^ In 1985−86, staff of the Cuban Institute of Cardiology (CIC) and the Pinar del Rio Teaching Hospital (PRTH) began the planning of the Pinar del Rio Project, a joint project for the prevention and control of RF and RHD in this province.

## RF and RHD prevention and control

It is well known that Group A streptococcal pharyngitis infections cause RF and RHD, and that the host’s genetic susceptibility to the bacterium as well as his/her socio-economic environment influence the spread of the disease.^14^ Currently, there are five major impediments to overall improvement in prevention and control of RF and RHD:

● shortage of an anti-streptococcal vaccine
● economic constraints such as poverty, under-nutrition, overcrowding and poor housing
● limited expertise of healthcare providers in prevention and management of RF and RHD, as well as shortage of human and material resources, limited supplies of penicillin and limited accessibility to health centres
● low levels of awareness and involvement of the healthcare system, patients, their families and the community; and sometimes social taboos against the use of penicillin
● poor adherence of patients to secondary prophylaxis. We report below how the Pinar del Rio project was planned and implemented, and present the results of the 15-year plan.

## Objectives

The overall goal was to reduce morbidity, disability and mortality caused by RF/RHD and its complications. The specific goals were: to develop and implement a practical strategy for community-based prevention and control of RF/RHD; to evaluate the applicability, effectiveness, cost and benefit of the methods (1986−1996); to assess the viability of the programme after the project period was over (1997−2002).

## Methods and project design

The project was designed as a service-oriented plan to be implemented through the primary healthcare (PHC) structure and facilities of the national health system, supported by the education system, with the participation of hospitals, primary healthcare units, schools, teachers, patients, their families and the public. The project concentrated efforts on three of the more easily rectified impediments, namely, limited expertise of healthcare providers, low level of public awareness of the disease, and poor adherence of patients to prophylaxis.

The programme was implemented in Pinar del Rio Province in the western part of Cuba, and included all five- to 25-year-olds with permanent residence in the province during the period January 1986 to December 1996 and January 1997 to December 2001. It involved primary and secondary prevention of RF/RHD, training of health personnel, healthcare education via dissemination of information, community involvement and epidemiological surveillance.

**Fig. 1.**
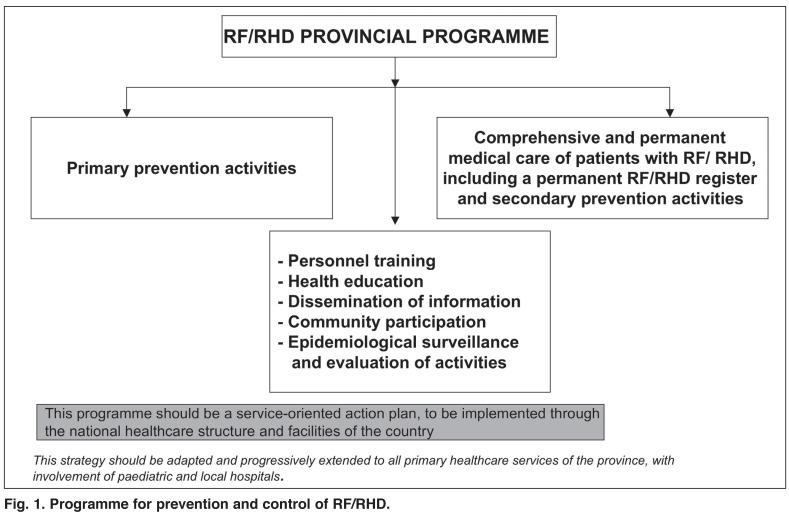
Programme for prevention and control of RF/RHD.

Two cross-sectional studies were carried out on the prevalence of RF/RHD in school children. The programme advisory committee consisted of a part-time provincial cardiologist, supported by representatives of related departments, such as paediatrics, cardiology, primary healthcare, hospital care and epidemiology, as well as microbiology laboratories, nurses and a representative of the Ministry of Education. There was one collaborating physician as local representative in each hospital (Fig. 1). An evaluation of the implementation of the project was done five years later.

A permanent provincial RF/RHD register was established, with a central register in the PRTH and six local registers in hospitals, which were closely connected with primary healthcare centres, policlinics and family physicians in the province. The register centres conducted registration, follow-up and secondary prophylaxis, and collected the standardised registration forms for monthly forwarding to the provincial register. Echocardiography was used for diagnosis and determining the severity of carditis and RHD.

Primary prevention activities were based on early and correct diagnosis and treatment of sore throats and streptococcal pharyngitis (strep-throat). Secondary prevention involved case finding, referral, registration, surveillance, follow-up and regular secondary prophylaxis for RF and RHD patients. Criteria and procedures for all these activities were described in previous publications.^13^,^15^,^18^,^20^-^24^

Training of healthcare and educational personnel, mainly staff of local hospitals and primary healthcare units was carried out through provincial, local and health unit workshops, seminars or continuing health education courses. These were organised by the provincial official responsible (PR), members of the programme advisory committee (PAC) or trained doctors of local hospitals.

The main content of these activities was early detection and treatment of sore throats and streptococcal pharyngitis, and referral of any possible RF and RHD patients, the importance of effective treatment and follow-up, monitoring compliance with secondary prophylaxis, measures for dealing with non-compliance cases, procedures for penicillin skin tests and treatment of anaphylactic reactions, and prevention of infectious endocarditis. At the end of each training activity, we distributed educational brochures and posters on the subject. Initially we distributed educational material prepared by the CIC, and later the Spanish version of the RF/RHD booklets provided by the WHO/ISFC/UNESCO joint project for RF/RHD prevention .

Healthcare educational activities were directed at the general population and schools, including teachers, school children and parents, as well as face-to-face interviews with patients and relatives. Provincial radio and television channels were also used to disseminate information. All activities included aspects of RF and RHD, stressing procedures for prevention, correct diagnosis and treatment of sore throats and strep-throats, and the adherence of RF/RHD patients to secondary prophylaxis.

Two cross-sectional studies of RF/RHD were carried out on school children (five- to 15-year-olds), one at the beginning (1985) and the other at the end of the study period (1996). The sample included school children enrolled at all schools in each municipality of the province, including urban, suburban and rural areas, at all socio-economical levels, during the period of a year. We used the multi-stratified random-sample method.

During the 10-year period, we had permanent surveillance of RF/RHD morbidity and mortality, the work of the register centres, the activities of healthcare and educational units, and compliance with the plan of operation. The PR made a brief evaluation of programme activities at provincial and local levels five years after the study period.

The estimated direct costs of RF/RHD care during the period were determined and evaluated, with the collaboration of the Department of Economy and medical assistance of the province. The PRTH controlled the training and educational activities, as well as the supply of penicillin and reagents. Data for each of the approaches were collected on the specific data collection forms and later processed and analysed using the EPI-INFO 5 statistical software and presented in the form of tables and graphs. Whenever needed, the χ2 statistical test was performed.

## Results

Pinar del Rio is a Cuban province of 10 931 km2, located in the western part of the Island with a population in 1996 of 721 800 inhabitants, 62.5% of whom lived in urban areas.29 It has a well-structured medical assistance system with free and easy accessibility to medical care and treatment for the whole population.

Education and training were carried out at provincial, local and health unit level as part of the normal continuing medical education of the province (one to three per year), with participation of invited lecturers and the involved physicians and nurses. Advertisements and educational information about the project were broadcast on provincial radio and television channels two to four times per year.

There was a progressive decline in the occurrence and severity of RF and RHD in the whole province during the programme, with a marked decrease in prevalence of inactive RF (a patient with a history of acute RF, but without evident heart valve damage) and RHD (a patient with or without a history of acute RF, but with typical rheumatic heart valve damage, supported by an echocardiogram). The decline in RF was from 8.0 cases per 1 000 school children in 1985 to 2.0 per 1 000 in 1996, and a decline in RHD from 2.2 to 0.24 per 1 000 (10.5 times lower) (Table 1). There was also a marked decrease in the severity of RHD; in 1986 there were five cases (38.5 %) of severe RHD, whereas in 1996 only one was reported (16.7%).

**Table 1.** Prevalence Of RF And RHD In Five- To 14-Year-Olds. Pinar Del Rio, Cuba

*1985 (n 5 5 708)*		*1996 (n 5 25 519)*	
*Type of RF/RHD*	*No of confirmed patients (rate per 1 000)*	*Type of RF/RHD*	*No of confirmed patients (rate per 1 000)*
History of acute RF without established RHD	33 (1.75)	History of acute RF without established RHD	43 (5.78)
Established RHD*	13 (2.27)	Established RHD*	6 (0.24)
Total**	46 (8.01)	Total**	49 (1.99)

*χ^2^ 5 31.92; *p* < 0.000001; **χ^2^ 5 57.38; *p* < 0.000001

**Fig. 2.**
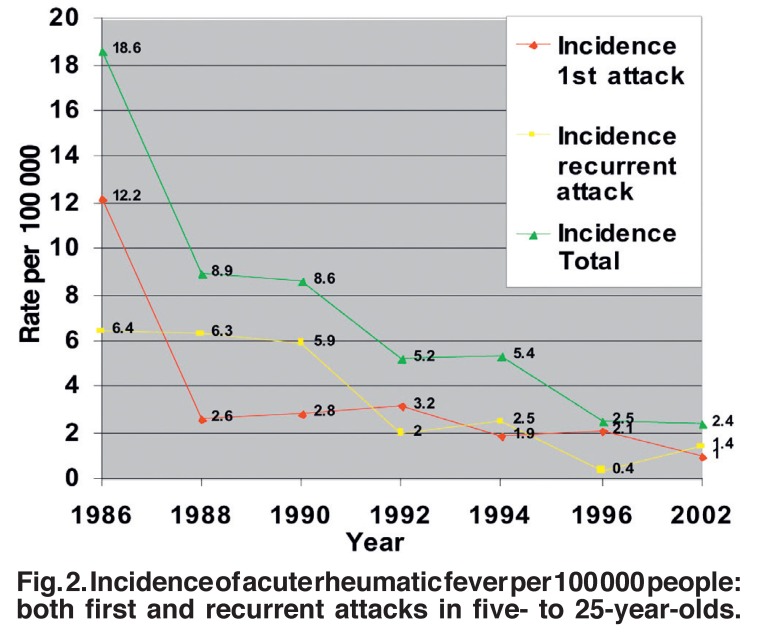
Incidence of acute rheumatic fever per 100 000 people: both first and recurrent attacks in five- to 25-year-olds.

The incidence per 100 000 children of school-going age of both first and recurrent acute attacks of RF also had a marked decrease from 28.4 per 100 000 in 1986 to 2.7 in 1996, most markedly in the incidence of a first attack. This was also observed when we included the age group five to 25 years old (Table 2, Fig. 2).

**Table 2.** Incidence Of RF And RHD In Pinar Del Rio, Cuba

**Incidence in five- to 25-year-olds**						
	*1986*		*1996*		*2002*	
*Attack*	*Population (279 400)*	*Rate per 100 000*	*Population (233 898)*	*Rate per 100 000*	*Population (207 815)*	*Rate per 100 000*
First	34	12.2	5	2.1	2	1.0
Recurrent	18	6.4	1	0.4	3	1.4
Total*	52	18.6*	6	2.5*	5	2.4
**Incidence in five- to 14-year-olds**						
	*1986*		*1996*		*2002*	
*Attack*	*Population (119 600)*	*Rate per 100 000*	*Population (106 302)*	*Rate per 100 000*	*Population (104 375)*	*Rate per 100 000*
First	28	23.4	2	1.8	2	1.9
Recurrent	6	5.0	1	0.9	1	0.9
Total*	34	28.4**	3	2.7**	3	2.8

*χ^2^ = 29.01; *p* < 0.000001; **χ^2^ = 22.5; *p* < 0.000002

In 1986, 36.6% of patients had carditis, 2.8% chorea and 59.6 % arthritis, while in 1996 only one patient (16.7%) had carditis. In 1986, 30.8% of the 52 acute attacks had heart valve sequelae, which were higher and more severe among those with recurrent attacks (38.8%), whereas in 1996 there were only six acute cases, and only one with sequelae, a mild mitral regurgitation. There was also a progressive decline in the number and severity of patients requiring hospitalisation after the acute attack, from 41.1% of the 134 registered cases during 1986−90 (11.2% for heart failure and 4.5% for heart valve surgery) to 8.3% of the 193 registered during 1991−96 (1.5% for heart failure and 0.5% for heart valve surgery). During 1996 there was a total of 193 registered patients; only one required hospitalisation (0.5%) and none for heart failure or heart valve surgery. There were no patients with infectious endocarditis during the study period (Table 3).

**Table 3.** Patients Requiring Hospitalisation After The Acute Attack* (All Registered Patients 5−25 Years Old)

				*RHD patients requiring one or more hospitalisation after the acute attack*							
*Year*	*Number of registered patients (mean of new patients per year)*	*Patients not requiring hospitalisation*		*Total*		*Without heart failure*		*With heart failure*		*Requiring heart valve surgery*	
n	%	n	%	n	%	n	%	n	%
1986–90	134 (27 per year)	79	58.9	55	41.1	34	25.4	15	11.2	6	4.5
1991–96	193 (10 per year)	177	91.7	16	8.3	12	6.2	3	1.5	1	0.5
1986*	52	39	75.0	13	25.0*	7	13.4	5	9.6	1	1.9
1996*	193	192	99.5	1	0.5*	1	0.5	0	.	0	.

* χ^2^ = 36.15; *p* < 0.00001 *All patient with acute RF or symptomatic RHD are hospitalised (in local hospital or in home care) or referral to high-tech hospital.

Compliance with secondary prophylaxis increased progressively during implementation of the project. In 1986, among the 52 registered patients, the compliance was 50% regular, 36.5% irregular and 13.5% non-compliance, with a high percentage of recurrent attacks in both irregular-compliance (63.2%) and non-compliance (57.1%) patients, whereas in 1996, of the 193 registered patients, 93.8% were following regular secondary prophylaxis and 6.2% irregular prophylaxis, with only one recurrent attack among the irregular-compliance group.

**Fig. 3.**
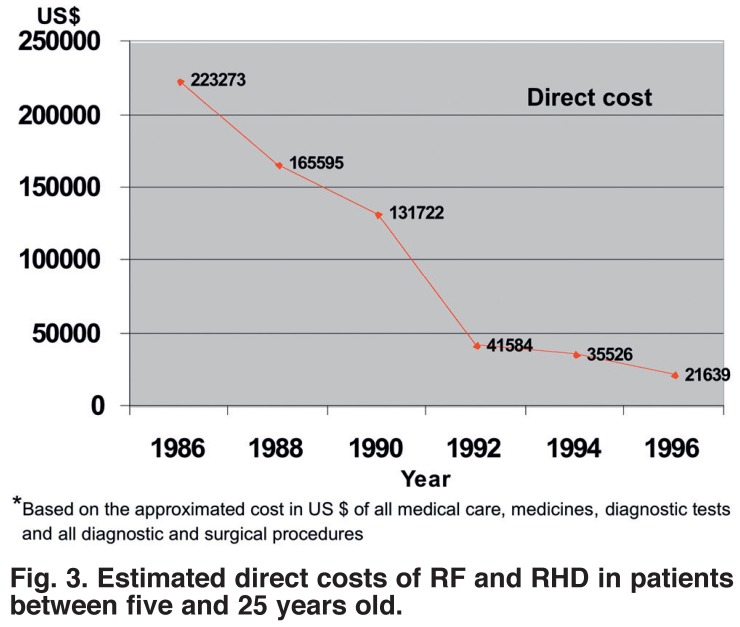
Estimated direct costs of RF and RHD in patients between five and 25 years old.

The estimated direct costs of RF/RHD care in the province decreased progressively, with a mean cost of 97 457.00 US $ per year during the 10 years of the project, 145 519 US $ per year between 1986 and 1990, and 49 376 US $ from 1991 to 1996, with only 21 639 US $ during 1996 (Fig. 3).

The implementation of the programme did not involve additional costs for staff, because the PR and local representatives managed the project during normal working hours. Since the project was run as part of the normal heathcare system and the number of RF/RHD patients was not too large, it did not inconvenience the healthcare system in the province and therefore ensured successful implementation. The additional cost was approximately 2 000 US $ per year, mainly for preparing, editing and printing medical information, and for educational activities, but rarely for the supply of B penicillin and reagents. The programme ensured more efficient use of antibiotics, particularly B penicillin, and there was no increased use of antibiotics.

At the end of the project in 1996, RF/RHD remained one of the chronic registered diseases for follow-up at local and primary healthcare services in the province. A brief report on RF/RHD prevention activities in school children in the province in 2002 showed that referral of patients to local hospitals and the implementation of primary and secondary prophylaxis remained ongoing, with more than 80% of RF/RHD patients regularly complying with secondary prophylaxis. A few cases were confirmed, with an incidence of first attack of 1.0 case per 100 000 in five- to 25-year-olds, recurrences of 0.9 per 100 000, and a total of 2.4 cases per 100 000 (Table 2). There were no reported RHD patients either needing heart valve surgery or with infectious endocarditis, and no deaths were reported.30

## Discussion

Although the number of cases of RF and RHD was not very high at the start of the programme, it was very opportune of health authorities to include RF/RHD in the healthcare priorities of the province. Training of personnel and the educational activities were well implemented and the staff at PHC and local hospitals was more competent and knowledgeable, as were patients and their families. This ensured a more timely diagnosis of sore throats and strep-throats and more efficient use of antibiotics.

Our results show that the involvement of local hospitals and primary healthcare systems in our programme increased the medical awareness of young patients, who were the ones to traditionally show high mortality and disability rates.^9^,^12^,^14^ The continued low incidence and severity of the disease five years after the project ended was confirmation that staff and patients remained aware of RF/RHD, and particularly that RF/RHD remains one of the chronic registered diseases for follow-up at local hospitals and primary healthcare services in the province.^30^

The marked decline in the direct costs of managing the disease (86.1%) was significant, due principally to the smaller number of cases and less severe disease, with fewer hospitalisations and no cases requiring heart surgery in the later years.

The implementation of training and educational activities was included in the normal provincial continuing medical education system and healthcare education of the population, hence it did not require additional budget, only a small amount for the preparation of educational material and activities. There was no need for additional budgets for staff and facilities for the implementation of preventive measures, control and surveillance of the disease, because these were included as part of the normal structure of the healthcare system. We should highlight the high level of interest and responsibility of the selected responsible officers, and the contribution of the teaching units.

The extension of the family physician’s service could have helped in the successful implementation of the project. However, we assumed that the project strategy applied, particularly the training of healthcare personnel and dissemination of educational material and posters, played the most important role, as was found in the report of Bach *et al.* in the two French Caribbean islands.^19^ The support of health authorities and hospitals, and the involvement of staff and patients also contributed to the success of the project.

## Conclusions and recommendations

● Prevention and control of RF/RHD is feasible and affordable in developing countries, by adapting WHO recommendations to the healthcare system and facilities of specific areas or provinces.
● A preliminary protocol with the basic components of the project would enable understanding by policy makers and potential donors.
● Obtaining approval and support of high-level authorities and teaching institutions ensures the participation of different levels of the healthcare system and the solution of most problems in this regard.
● The training of healthcare personnel, health education for the population, and the dissemination of simple posters and educational material at least once or twice a year play an important role in the successful implementation of the programme.
● Prevention and control of RF/RHD is cost-effective and does not increase antibiotic usage, but promotes effective use of them.
● The implementation of the programme as part of the normal healthcare system’s structure and facilities decreases the budget requirements and ensures the continuation of activities several years after the project ends.
● Participation in joint national or international projects encourages staff and responsible officers to collaborate and write up reports.

## References

[1] Mayosi B, Robertson K, Volmink J, Adebo W, Akinyore K, Amoah A (2006). The Drakensberg declaration on the control of rheumatic fever and rheumatic heart disease in Africa.. S Afr Med J.

[2] Roberson KA, Volmink JA, Mayosi BM (2006). Towards a uniform plan for the control of rheumatic fever and rheumatic heart disease in Africa − The Awareness, Surveillance, Advocacy, Prevention (ASAP) programme.. S Afr Med J.

[3] Clur S-A (2006). Frequency and severity of rheumatic heart disease in the catchment area of Gauteng hospitals, 1993−1995.. S Afr Med J.

[4] Carapetis JR, McDonald M, Wilson (2005). Acute rheumatic fever.. Lancet.

[5] Carapetis JR, Steer AC, Mulholland EK, Weber M (2005). The global burden of Group A streptococcal disease.. Lancet Infect Dis.

[6] Longo-Mbenza B, Bayekula M, Ngiyulu R, Kintoki VE, Bikangi NF, Seghers KV (1999). Survey of rheumatic heart disease in school children of Kinshasa town.. Int J Cardiol.

[7] Steer AC, Carapetis JR, Nolan TM, Shann F (2002). Systematic review of rheumatic heart disease prevalence in children in developing countries. The role of environmental factors.. J Paediatr Child Hlth.

[8] Report of a WHO study group..

[9] Taranta A, Markowitz M (1989). Rheumatic Fever.. Boston: Kluwer Academic.

[10] Kaplan E (1996). Recent epidemiology of Group A streptococcal infections
in North America and abroad: an overview.. Pediatrics.

[11] (1997). Conquering Suffering. Enriching Humanity.. Geneva, World Health Organisation.

[12] Kumar RK, Rammohan R, Narula J, Kaplan E, Narula J (1999). Epidemiology of streptococcal pharyngitis, rheumatic fever and rheumatic heart disease.. Rheumatic Fever..

[13] Joint WHO/ISFC meeting on RF/RHD control with emphasis on primary prevention.

[14] Rheumatic Fever and Rheumatic Heart Disease. Report of a WHO Expert consultation..

[15] WHO global programme for the prevention of RF/RHD. Report of a consultation to review progress and develop future activities..

[16] Arguedas A, Mohs E (1992). Prevention of rheumatic fever in Costa Rica.. J Pediat.

[17] Gordis L (1985). The virtual disappearance of rheumatic fever in the United States: lessons in the rise and fall of disease.. Circulation.

[18] Strasser T, Dondog N, El Kholy A, Gharagozloo R, Kalbian VV, Ogunbi O (1981). The community control of rheumatic fever and rheumatic heart disease: report of a WHO international cooperative project.. Bull Wld Hlth Org.

[19] Bach JF, Chalons S, Forier E, Elana G, Jouanelle J, Kayemba S (1996). Ten-year educational programme aimed at rheumatic fever in two French Caribbean islands.. Lancet.

[20] Nordet P, Rojas J, Lopez R (1988). Fiebre reumática in Ciudad de la Habana, 1972−1982.. Incidencia y caracteristicas. [Rheumatic fever in Havana. Incidence and characteristics, 1972–1982.] Revista Cubana Pediatria, [Cuban J Pediat].

[21] Nordet P, La Llave G, Lopez H, Aranguren P, Lopez R (1989). Fiebre reumática in Ciudad de la Habana. Prevalecía y características, 1972–1987.. [Rheumatic fever in Havana. Prevalence and characteristics, 1972–1987.] Revista Cubana Pediatría, [Cuban J Pediat].

[22] Nordet P, Rojas J, la Llave G, Lopez R, Alfonso A, Rodríguez L (1991). Fiebre reumática en Cuba: incidencia, prevalecía, mortalidad y características clínicas.. [Rheumatic fever and rheumatic heart disease in Cuba: incidence, prevalence mortality and clinical characteristics.] Revista Cubana de Cardiología y Cirugía Cardiovascular, [Cuban J Cardiol Cardiovasc Surg].

[23] López RR Fiebre Reumática. Estudio Clínico-epidemiológico. Provincia de Pinar del Río, Cuba. 1986−1996..

[24] (1992). WHO programme for the prevention of rheumatic fever/rheumatic heart disease in 16 developing countries (AGFUND). Report from Phase I (1986−1990).. Bull Wld Hlth Org.

[25] Zuazo J, Nordet P, Soto N, Suarez M (1980). Infección estreptocócica en una población escolar primaria. Estudio durante un curso escolar.. Rev Cubana Med Trop.

[26] Nordet P, Zuazo J, Leon A (1978). Estudio clínico epidemiológico de la frecuencia de infección estreptocócica en niños con amìgdalo faringitis aguda.. Rev Cubana Higiene Epidemiol.

[27] Nordet P, Zulueta JM, Navarro E, Marin CA (1989). Amigdalofaringitis aguda. Estudio clínico-epidemiológico y terapéutico. [Acute tonsilo-pharyngitis. Clínicas, bacteriológicas and therapeutic study.]. Rev Cubana Pediat [Cuban J Pediat].

[28] Nordet P, Alfonso A, López R (1988). Fiebre Reumática. Estado clínico y evolución a los 5 y 10 años del primer ataque.. Rev Cubana Pediat.

[29] Anuario Estadístico de Salud. Estadísticas de Salud en Cuba. MINSAP, La Habana, Cuba. 2004.. [Ministry of Public Health. Annual Statistic of Health. Health Statistics in Cuba. MINSAP, Havana, Cuba, 2004.].

[30] (2003). [Rheumatic Fever. Incidence and clinical-epidemiological characteristics in the Pinar del Rio Province, Cuba. 2002. Thesis for specialisation in Internal Medicine.]. Hospital Provincial León Cuervo Rubio, Pinar del Río, Cuba.

